# Obesity-Associated Oxidative Stress: Strategies Finalized to Improve Redox State

**DOI:** 10.3390/ijms140510497

**Published:** 2013-05-21

**Authors:** Isabella Savini, Maria Valeria Catani, Daniela Evangelista, Valeria Gasperi, Luciana Avigliano

**Affiliations:** Department of Experimental Medicine and Surgery, University of Rome Tor Vergata, Via Montpellier 1, 00133 Rome, Italy; E-Mails: catani@uniroma2.it (M.V.C.); d.evangelista@med.uniroma2.it (D.E.); gasperi@med.uniroma2.it (V.G.); avigliano@uniroma2.it (L.A.)

**Keywords:** oxidative stress, obesity, cardiovascular disease, cancer, diet, phytochemicals, vitamins, physical activity

## Abstract

Obesity represents a major risk factor for a plethora of severe diseases, including diabetes, cardiovascular disease, non-alcoholic fatty liver disease, and cancer. It is often accompanied by an increased risk of mortality and, in the case of non-fatal health problems, the quality of life is impaired because of associated conditions, including sleep apnea, respiratory problems, osteoarthritis, and infertility. Recent evidence suggests that oxidative stress may be the mechanistic link between obesity and related complications. In obese patients, antioxidant defenses are lower than normal weight counterparts and their levels inversely correlate with central adiposity; obesity is also characterized by enhanced levels of reactive oxygen or nitrogen species. Inadequacy of antioxidant defenses probably relies on different factors: obese individuals may have a lower intake of antioxidant- and phytochemical-rich foods, such as fruits, vegetables, and legumes; otherwise, consumption of antioxidant nutrients is normal, but obese individuals may have an increased utilization of these molecules, likewise to that reported in diabetic patients and smokers. Also inadequate physical activity may account for a decreased antioxidant state. In this review, we describe current concepts in the meaning of obesity as a state of chronic oxidative stress and the potential interventions to improve redox balance.

## 1. Introduction

Obesity is a serious nutritional problem, as it increases the risk of morbidity from several pathologies, including hypertension, dyslipidemia, type 2 diabetes, coronary heart disease, stroke, non-alcoholic fatty liver disease, osteoarthritis, sleep apnea, and endometrial, breast, prostate, and colon cancers [1]. Obesity is defined as excessive fat accumulation that may impair health and increase mortality (WHO, 2012); the current classification of obesity is based on the Body Mass Index (BMI), which is the weight (in kilograms) divided by the square of height (in meters). Definition of BMI “normal range” is based on Caucasian mortality data; a BMI of 30 kg/m^2^ or more is index of obesity, while a BMI over 25 kg/m^2^ is index of overweight [1]. For Asian populations, a BMI of 27.5 kg/m^2^ or more is index of obesity whereas a BMI over 23 kg/m^2^ is index of overweight. BMI has limitations because it does not distinguish between lean mass and fat; it may overestimate body fat in well-trained body builders and underestimate body fat in older persons; moreover, BMI does not identify fat distribution. It is now well recognized that abdominal fat is a major risk for obesity-related diseases: indeed, visceral fat accumulation contributes to pro-oxidant and pro-inflammatory states, as well as to alterations in glucose and lipid metabolisms [1,2]. Waist circumference (WC) or waist-to-hip ratio (WHR) are useful indicators of visceral fat distribution: WC equal to or more than 80 cm (in women) or 94 cm (in men) and WHR above 0.90 for males and 0.85 for females are associated to high cardiometabolic risk in Europeans [3]. However, these cut offs may not be appropriate for African-American, Hispanic, and Middle Eastern populations [4].

Excessive fat accumulation is a consequence of positive energy balance that results from interaction among several factors, including diet (increased intake of energy-dense foods and decreased intake of food rich in micronutrients and bioactive compounds) [5], decreased physical activity (sedentary lifestyle), nutritional and hormonal status in early life [6], as well as genetic, environmental, cultural, and economic factors [7,8]. Other etiological factors that are associated with obesity are some chromosomal aberrations (such as Prader-Willi syndrome), hormonal pathologies (such as Cushing’s disease), hypothalamic lesions or tumors, and drugs (such as steroids and antidepressants) [9–11].

Epidemiological, clinical, and animal studies have shown that obesity is coupled with altered redox state and increased metabolic risk [12–17]. Oxidative stress can be a consequence, but also a trigger of obesity. Chronic hypernutrition, high fat high carbohydrate (HFHC) meals, as well as high dietary saturated fatty acids (SFA) and trans-fatty acids, stimulate intracellular pathways, leading to oxidative stress through multiple biochemical mechanisms, such as superoxide generation from NADPH oxidases (Nox), oxidative phosphorylation, glyceraldehyde autoxidation, protein kinase C (PKC) activation, and polyol and hexosamine pathways [18–20]. Oxidative stress could play a causative role in the development of obesity by stimulating white adipose tissue deposition and altering food intake: cell culture and animal studies show that oxidative stress increases pre-adipocyte proliferation, adipocyte differentiation and size of mature adipocytes [21–23], and reactive oxygen species (ROS) seem to be involved in the control of body weight, by exerting different effects on hypothalamic neurons that control satiety and hunger behavior [24]. It has also been demonstrated that obesity *per se* can induce systemic oxidative stress: indeed, fat accumulation increases Nox activity and endoplasmic reticulum (ER) stress in adipocytes that lead to increased ROS production [21,25]. Other factors that contribute to oxidative stress in obesity are abnormal post-prandial ROS generation [26], hyperleptinemia [27], chronic inflammation [28], tissue dysfunction [20], and low antioxidant defenses [29,30]. Oxidative stress and inflammation appear to be closely interlinked in obesity, although it is difficult to establish the temporal sequence of their relationship. For example, several pro-inflammatory transcription factors, including nuclear factor-κB (NF-κB) and activator protein-1 (AP-1), are redox sensitive; therefore, ROS trigger the release of inflammatory cytokines, which in turn enhance ROS production [31], thus establishing a vicious circle. Systemic oxidative stress and inflammation are also key factors in the pathogenesis of obesity-related diseases, including atherosclerosis, insulin resistance, type 2 diabetes, and cancer [32,33]. Recently, it has been suggested that increased oxidative stress and inflammation in obesity also enhance aging processes [34].

Strategies to lower oxidative stress in obesity include weight loss, physical activity, and antioxidant-rich diet. It is known that weight reduction decreases oxidation markers, increases antioxidant defenses and improves metabolic and cardiovascular risks associated with human obesity [35]. It is well established that a diet abundant in fruits, vegetables, whole grains, legumes, fish, olive oil, and dairy fermented foods is helpful to maintain weight and reduce the incidence of metabolic diseases [36]. Beneficial components present in these foods are some macronutrients (such as monounsaturated fatty acids (MUFA) and ω-3 polyunsaturated fatty acids (ω-3 PUFA)), vitamin C, vitamin E, phytochemicals, and probiotics [37–39]. *In vitro* and *in vivo* studies show that these dietary factors can act trough several mechanisms, such as cell signaling and modulation of gene expression, reduction of obesity-induced oxidative stress, production of inflammatory molecules, and lipid accumulation [40,41]. Despite these effects, data from observational and human intervention studies are controversial and failed to demonstrate that addition of a single dietary component reduces obesity or obesity-associated pathologies [42–44]. It is, therefore, likely that healthy effects observed with consumption of such foods may be ascribed to cumulative effects of multiple nutrients.

The present study aims to review current concepts in the meaning of oxidative stress in human obesity and potential strategies finalized to maintain a correct redox balance, especially focusing on natural approaches (including weight loss, physical activity, diet, dietary supplementation, and microbiota modulation) rather than pharmacological treatments or surgical intervention.

## 2. Redox Balance in Obesity

Reactive oxygen (ROS) and nitrogen (RNS) species include superoxide (O_2_^·−^), hydrogen peroxide (H_2_O_2_), hypochlorite (ClO^−^), hydroxyl radical (OH^·^), nitric oxide (NO), and peroxynitrite (ONOO^−^). In physiological conditions, mitochondria are the major site of intracellular ROS production, due to electron leakage along the respiratory chain; nevertheless, they can also arise from plasma membrane systems, endoplasmic reticulum, lysosomes, peroxisomes and cytosolic enzymes. At low concentrations, ROS/RNS exert a multitude of biological effects, including immune-mediated defense against pathogenic microorganisms and intracellular signaling; conversely, high levels of these extremely reactive species can damage DNA, lipids, and proteins, thus leading to tissue injury and cell death [45].

To keep ROS/RNS correct levels, tissues possess antioxidant molecules working in synergy to minimize free radical cytotoxicity. Endogenous antioxidant compounds are urate, glutathione, ubiquinone, and thioredoxin; furthermore, some proteins (ferritin, transferrin, lactoferrin, caeruloplasmin) act as antioxidants, as they bind and sequester transition metals that may start oxidative reactions. Antioxidant enzymes are superoxide dismutase (SOD), glutathione peroxidase (GPx), glutathione reductase, glutathione *S*-transferase, catalase, thioredoxin reductase, peroxiredoxins (Prx), and NAD(P)H:ubiquinone oxidoreductase (NQO1). Recently, the paraoxonase (PON) family has emerged as a new class of antioxidant enzymes, playing an important role in obesity-associated illnesses, including cardiovascular disease (CVD) and diabetes mellitus; in particular, being present on the surface of high density lipoproteins (HDL), PON1 protects low density lipoproteins (LDL), and circulating cells against oxidative damage, thus preventing inflammatory responses in arterial wall cells. In addition, heme oxygenase-1 (HO-1), the rate-limiting enzyme in heme metabolism, can be considered an antioxidant enzyme able to reduce oxidative stress and inhibit inflammation. Recent results indicate that HO-1 plays beneficial roles in CVD, and in regulation of body weight and metabolism in diabetes and obesity. Dietary antioxidants include vitamin C, vitamin E, and a broad spectrum of bioactive compounds (such as phytochemicals). Zinc, manganese and selenium should be regarded as crucial nutrients for activity of antioxidant enzymes: SODs use either manganese (Mn-SOD) or copper and zinc (Cu-, Zn-SOD), whereas GPx1-4 and GPx6 are selenium-containing enzymes.

### 2.1. Assessment of Redox State

In humans, redox balance is generally evaluated by measuring markers of antioxidant defense and/or oxidative stress. Plasma concentrations of molecules (retinol, carotenoids, vitamin E, vitamin C, glutathione, uric acid), minerals (especially selenium and zinc), as well as antioxidant enzyme activities are the most widely used biomarkers of the antioxidant state. Another useful biomarker is the total antioxidant capacity (TAC) that evaluates the integrated action of all antioxidants present in plasma [46]. An emerging approach is the quantitative proteomic method allowing simultaneously quantification of changes that take place in different members of the antioxidant enzyme network [47].

Oxidative stress can be evaluated by direct assessment of free radical production (by electron spin resonance (ESR) or immuno spin-trapping methods) [48,49] or by indirect methods that assess end-products of oxidative damage to proteins and aminoacids (such as protein carbonyls, 3-nitrotyrosine, advanced glycosylation end products (AGEs) and advanced oxidation protein products (AOPPs)) [50], lipids (F2-isoprostanes, malondialdehyde (MDA), oxidized LDL (oxLDL), thiobarbituric acid reactive substances (TBARs) and 4-hydroxynonenal (4-HNE)) [51] and nucleic acids (such as 8-hydroxy-2′-deoxyguanosine (8-OHdG)), in blood and urine [45]. Another method that has been widely used to investigate oxidative stress in various diseases and in post-prandial responses is ROS generation by isolated leukocytes [19].

### 2.2. Association between Oxidative Stress and Obesity

High fat deposition is strictly related to redox unbalance. Juvenile overweight and obesity have been linked to high levels of oxidative stress [12,13]; obese subjects show higher oxLDL, AAOPs and TBARs levels than control subjects [52]. In adults, BMI, total body fat, and waist circumference have been shown to be positively correlated with urinary F2-isoprostane levels and inversely correlated with PON1 activity [53–55]. Interestingly, F2-isoprostane levels may predict loss of total adiposity over time: a strong significant inverse association between urinary F2-isoprostanes and weight gain, during a five-year follow-up period, was demonstrated by both the Insulin Resistance Atherosclerosis Study (299 participants) [56] and the Health, Aging, and Body Composition Study (726 participants) [57]. This inverse association has been interpreted as a positive physiological response to address excess adiposity and/or a catabolic response to inflammation. In mice, diet-induced obesity increases cerebrocortical oxidative stress [58] and high fat diet-induced obesity also correlates with mitochondrial dysfunction and increased oxidative stress in skeletal muscle and liver [59]. Hearts from rodents maintained on high fat diet display increased indices of lipid and protein oxidation, as well as increased markers of apoptosis [60].

Despite the strong association between obesity and oxidative stress, none of the above mentioned markers is a predictor of obesity development, but instead these biomarkers can predict development and progression of metabolic and CVD in overweight and obese people. A positive correlation between oxidative stress markers and markers of inflammation, hyperglicemia, and hyperlipidemia has been found. In young overweight and obese subjects, AOPPs positively correlate with central obesity, triglycerides, and insulin, and negatively correlate with glucose to insulin ratio and HDL-cholesterol, suggesting an increased metabolic risk in this population [14,15]. Urinary F2-isoprostanes are positively associated with circulating pro-inflammatory cytokines, such as monocyte chemoattractant protein-1 (MCP-1) and interleukin-6 (IL-6) [61], which are predictors of diabetes and CVD [28,32,62]. High levels of serum 8-OHdG have been found in pre-diabetic subjects [63]. Increased serum AGEs levels predict total and CVD mortality in women with type 2 diabetes [64]. Recently, it has been reported that urinary F2-isoprostanes and plasma oxLDL are strongly influenced by central adiposity indicators (waist circumference or waist-to-hip ratio) more in women than in men, suggesting that change of the gynoid to android phenotype could lead to a steeper unfavorable redox state in young women compared to men [16,57].

Altered antioxidant defenses are also observed in obese people, [65,66] nonetheless, the relationship between BMI, body fat, and antioxidant defenses is still an open question; indeed, no correlation at all [67] or a link with obesity-related diseases, instead of obesity *per se*, have been described [68]. This is not unexpected considering that the different antioxidant enzymes act in a fine-tuned temporal order and at the onset of obesity, tissues try to counteract oxidative stress (induced by elevated circulating fatty acids) by increasing expression and activity of antioxidant enzymes, which are progressively depleted as obesity occurs.

Evidence of altered antioxidant defenses in obesity arises from several observational, clinical, and *in vitro* studies. In a population-based sample of 3042 adults from the Attica area in Greece, an inverse relationship between visceral fat and TAC, regardless of other variables (such as sex, age, smoking, physical activity, and dietary habits), has been found; this correlation was stronger for waist circumference with respect of BMI [29]. Accordingly, a study, performed to determine whether obesity exacerbates lipoprotein abnormalities and oxidative stress in older men or not, showed that TAC, and vitamins C and E were lower, while hydroperoxides and carbonyl proteins were higher, in young and old obese patients compared to their respective controls; moreover, oxidative stress was aggravated in older adults [17]. SOD, catalase and GPx activities have been found to be inversely related to BMI, both in obese children and adults [69–71]. In obese women, serum GPx activity was significantly increased after weight reduction [72]. On the other hand, it has been reported that severely obese patients with greater insulin resistance have greater GPx activity than control counterparts [73]. Several experimental and clinical trials have shown that serum PON1 activity is decreased in obese subjects; a decrease in PON1 activity seems positively correlated with BMI and inversely correlated with HDL levels [52]. A positive association between cytosolic (Prx1 and Prx2) and mitochondrial (Prx3 and Mn-SOD) antioxidant enzymes and leucocyte ROS/RNS generation has been demonstrated in ten lean subjects after repeated intake of HFHC meal (1800 kcal for four days); this study also showed that compensatory mechanisms triggered by meals seemed to be insufficient to prevent or reverse diet-induced oxidative stress, as indicated by a persistent increase in MDA cellular content [74]. *In vitro* studies showed that extracellular SOD expression increases after differentiation of mice 3T3-L1 pre-adipocytes and then decreases; it is also up-regulated in differentiated 3T3-L1 cells co-cultured with stimulated macrophages, suggesting that SOD may be stimulated to protect adipocytes from oxidative stress generated by infiltrated macrophages [75]. In mice, high fat feeding increases H_2_O_2_ production within the heart and rapidly up-regulates catalase, probably to protect mitochondria from oxidative damage [47].

Deficiencies in minerals and vitamins can also contribute to impaired antioxidant defenses [30,76]; poor selenium and zinc nutritional state has been reported in obese children and adolescents, especially in children with central adiposity [77,78], whereas morbidly obese patients show magnesium, selenium, iron, and zinc deficiencies [79]. Low levels of carotenoids, vitamin C and E, related to BMI increase, have been found in Europe, USA, and elsewhere [80–83]. The Coronary Artery Risk Development in Young Adults (CARDIA) study reported a strong inverse relationship between BMI and the sum of serum carotenoids (α-carotene, β-carotene, β-cryptoxanthin, zeaxanthin/lutein) [84]. In the National Health and Examination Survey (III), despite similar self-reported intakes of fruit and vegetable servings, obese children show lower serum β-carotene and α-tocopherol concentrations than normal weight children [30]. Aasheim and Bøhmer [85] found that the most obese patients have the most pronounced reductions in vitamin state, especially vitamins A, B_6_, C, D, and E. The multicenter prospective population study of diet and cancer in Europe (EPIC) showed that plasma vitamin C concentration was inversely related to central fat distribution, in the cohort of Norfolk (UK) [86]. Accordingly, in 72 healthy Italian obese subjects (BMI: 40 ± 7 kg/m^2^; mean age: 44 ± 10 years), we found that plasma TAC and vitamin C values were lower than those measured in lean subjects (TAC: 0.92 ± 0.1 *vs.* 1.50 ± 0.3 mM Trolox equivalents; vitamin C: 0.023 ± 0.01 *vs.* 0.075 ± 0.03 mM); these individuals also showed higher oxidized/reduced glutathione (GSSG/GSH) ratio (0.18 ± 0.09 *vs.* 0.07 ± 0.01). Obese adults with a history of weight fluctuations (measured by weight cycling index; WCI) show the most serious vitamin C deficiency (0.014 ± 0.07 mM).

### 2.3. Conditions Generating ROS/RNS

**Figure 1:**
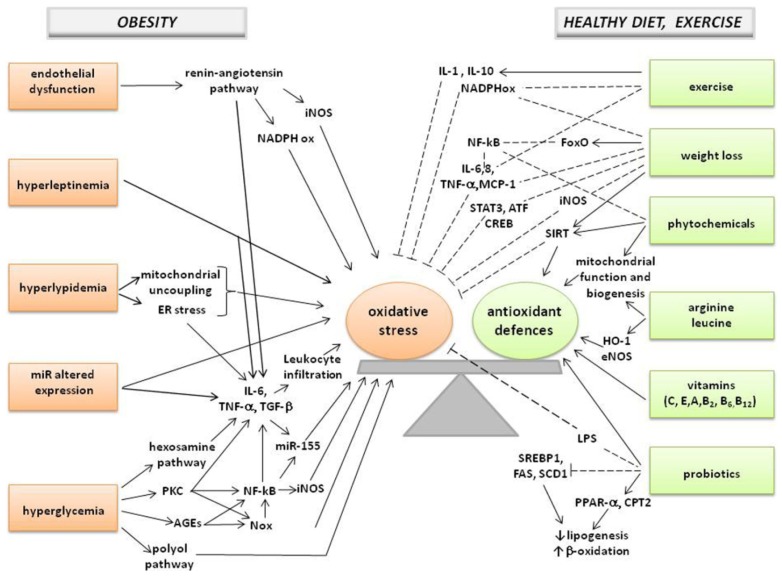
Mechanisms modulating oxidant/antioxidant balance in obesity. On the left-hand side are shown mechanisms underlying oxidative stress in obesity and obesity-associated complications, while on the right-hand side are shown strategies improving body antioxidant machinery (see text for details). Dotted lines: inhibitory effect. Solid lines: stimulatory effect. AGEs: advanced glycation end products; ATF: NF-κB, activating transcription factor; CPT2: carnitine palmitoyltransferase 2; CREB: cyclic AMP response element binding; ER: endoplasmic reticulum; FAS: fatty acid synthase; FoxO: forkhead box, sub-group O; HO-1: heme oxygenase-1; iNOS: inducible nitric oxide synthase; LPS: lipopolysaccharide MCP-1: monocyte chemotactic protein-1; miR: microRNA; NF-κB: nuclear factor-κB; Nox: NADPH oxidase; PKC: protein kinase C; PPAR-α: peroxisome proliferator-activated receptor-α; SCD1: stearoyl-CoA desaturase-1; SIRT: sirtuin; SREBP1: sucrose responsive element binding protein1; STAT3: signal transducer and activator of transcription 3; TGF-β: transforming growth factor-β; TNF-α: tumor necrosis factor-α.

Several mechanisms have been suggested to explain the enhanced oxidative stress observed in obese subjects, including altered lipid and glucose metabolism, chronic inflammation [28], tissue dysfunction [20], hyperleptinemia [27], and abnormal post-prandial ROS generation [26] (Figure 1).

When adipocytes reach non-physiological limits and become unable to function as an energy storage organ, lipotoxicity occurs. Fat is improperly accumulated in the heart, muscle, liver, and pancreas, where it exerts disruptive effects and triggers organ dysfunction. Intracellular triglycerides inhibit the adenosine nucleotide translocator (ANT), thus leading to ATP accumulation in mitochondria; the mitochondrial ADP drop reduces speed of oxidative phosphorylation and mitochondrial uncoupling promotes electron leakage and free radical release. In mouse skeletal muscle and liver, high fat diet-induced obesity triggers mitochondrial DNA damage [20]; this damage is associated with mitochondrial dysfunction, oxidative stress, and ER stress, characterized by impaired protein folding, lipid droplet creation, and hepatic cholesterol accumulation [59]. In physiological conditions, the oxidizing environment of ER favors formation of disulfide bonds and correct protein folding; during ER stress, misfolded proteins activate the “unfolded protein response” (UPR), responsible for ER biogenesis, protein folding and degradation of aberrantly packaged proteins. If UPR is prolonged, the persistent oxidative protein folding machinery causes ROS production, with subsequent systemic release of free fatty acids and inflammatory mediators [60]. Ectopic-fat deposition blocks glucose transport and insulin signaling: lipid accumulation in skeletal muscle has been associated with altered insulin sensitivity [87] and tissues from obese patients are resistant to the action of insulin [71].

Hyperglycaemia enhances the glycolytic pathway and the tricarboxylic acid (TCA) cycle, leading to NADH and FADH_2_ overproduction; the resulting increase in proton gradient across the mitochondrial inner membrane leads to electron leakage at complex III and superoxide production. The free radical, thus, inhibits the glyceraldehyde-3-phosphate dehydrogenase, thereby redirecting upstream metabolites into four alternative pathways: (i) glucose is shifted to the polyol pathway; (ii) fructose-6-phosphate is shifted to the hexosamine pathway; (iii) triose phosphates produce methylglyoxal, the main precursor of AGEs, while (iv) dihydroxyacetone phosphate is converted to diacylglycerol, thus activating PKC [88]. All these alternative pathways induce oxidative/nitrosative stress, by either enhancing free radical production or impairing antioxidant defenses. Indeed, glucose flux through the polyol pathway leads to NADPH depletion; glucosamine-6-phosphate generated in the hexosamine pathway inhibits thioredoxin activity and induces oxidative and ER stress; AGEs and PKC stimulate ROS/RNS production by activating Nox and NF-κB [89,90]. Nox enzymes catalyze the mono-electronic reduction of external oxygen using NADPH as an internal electron donor, thus producing superoxide; NF-κB drives transcription of adhesion molecules (including E-selectin, intercellular adhesion molecule-1 and endothelin-1), pro-inflammatory cytokines (including TNF-α and IL-6), iNOS and microRNAs (miR) involved in adipogenesis, inflammation and oxidative stress [91–93]. In particular, aberrant expression of specific miR observed in obese individuals may account for adipose hypertrophy and hyperplasia (especially miR-103, miR-143, miR-27a, and miR-27b), as well as for enhanced oxidative stress, inflammation and apoptosis (especially miR-155, miR-183, miR-221, miR-222, and miR-872) [91–93]. Finally, the hyperglycaemia/oxidative stress/inflammation axis is auto-perpetuating, as many inflammatory mediators impair insulin signaling [28], thus exacerbating hyperglycaemia and ROS production.

Tissue disfunction amplifies oxidative stress and inflammation by aggravating insulin resistance, hyperglycaemia, and hypertriglyceridemia. Indeed, increased expression of adipokines, (such as MCP-1, -2, -4, and macrophage inflammatory protein (MIP) -1α, -1β, -2α) causes macrophage infiltration in the adipose tissue, thus leading to overproduction of ROS and inflammatory cytokines [94]. Adipose tissue dysfunction also leads to adypocyte-specific deletion of nuclear factor E2-related factor 2 (Nrf2), a redox-sensitive transcription factor binding to the promoter region of genes coding for antioxidant enzymes (including NQO1 and proteins for glutathione synthesis), thus weakening antioxidant defenses [95]. In addition, endothelial dysfunction is a relevant phenomenon in obesity and obesity-induced hypertension: stimulation of the renin-angiotensin system, in obese individuals, activates Nox, xanthine oxidase and NOS. Elevated intraluminal pressure and hypertension itself increase ROS levels in the vasculature, thus enhancing endothelial dysfunction [96]. Angiotensin also regulates IL-6 and TNF-α secretion, thus, allowing monocyte recruitment in endothelium; the prolonged release of cytokines from these cells perpetuates inflammatory responses and aggravates vascular injury [97].

Leptin is a hormone produced by adipose tissue, which regulates appetite and exerts protective effects against lipotoxicity in non-adipose tissues. Hyperleptinemia induces oxidative stress, mainly by increasing mitochondrial and peroxisomal fatty acid oxidation [27,98]; it also stimulates proliferation and activation of monocytes/macrophages and production of IL-6 and TNF-α [99].

Another contributor to oxidative stress in obesity may be the altered post-prandial response to HFHC meals. Obese subjects show a more intense and prolonged oxidative and inflammatory stress (increased expression of p47phox subunit of Nox2, ROS generation and intranuclear NF-κB binding in mononuclear cells, as well as plasma MMP-9 concentrations) in response to a large HFHC meal than normal weight individuals [26]. Thus, chronic intakes of HFHC meals determine an even more negative effect in obese individuals.

## 3. Oxidative Stress in Obesity-Associated Diseases

Recent evidence suggests that oxidative stress may be the mechanistic link between obesity and co-morbidities, including non-alcoholic steatohepatitis, metabolic syndrome, type 2 diabetes, CVD, obstructive sleep apnea, and cancer.

### 3.1. Non-Alcoholic Fatty Liver Disease and Steatohepatitis

Obesity, hyperlipidemia and type 2 diabetes mellitus are frequently associated with non-alcoholic fatty liver disease (NAFLD), a form of liver dysfunction characterized by abnormal lipid accumulation in hepatocytes and liver inflammation. A serious consequence of NAFLD is non-alcoholic steatohepatitis (NASH) that can progress to liver fibrosis and cirrhosis [20].

NAFLD pathogenesis is not completely understood, especially because of its multifactorial origin and its dependence on some dietary factors, including fructose and certain types of fats [100]. Mitochondrial dysfunction, ER stress, and hyperglycaemia seem to be involved in NAFLD development trough oxidative stress. At the beginning of steatosis, carnitine palmitoyltransferase-1 (CPT1A) activity increases to compensate high fatty acid levels; at the same time, accelerated fatty acid catabolism causes excessive electron flux in the electron transport chain and ROS overproduction. The resulting oxidative stress alters mitochondrial morphology and function, thereby further increasing ROS generation [20]; then, by-products of lipid peroxidation generate 4-HNE-CPT1 adducts, thus inactivating CPT1. As a result, initially activated fatty acid catabolism is impaired and free fatty acids accumulate. ER stress triggers apoptosis via calcium perturbations, ROS production and activation of specific pro-apoptotic pathways by JNK-dependent signaling. Another contributor to lipid accumulation is hyperglycaemia; besides increasing oxidative stress as outlined in the previous paragraph, it activates the carbohydrate responsive element-binding protein (ChREBP), which transcriptionally modulates liver-type pyruvate kinase (L-PK) and all lipogenic genes [100].

### 3.2. Metabolic Syndrome

According to the International Diabetes Federation, metabolic syndrome (MS) is characterized by central obesity associated with two or more complications (hyperglycaemia, hypertension, dyslipidemia), whose co-existence represents an elevated risk factor for cardiovascular pathologies. The visceral fat area (more than the subcutaneous adipose tissue) has high levels of resident macrophages and cytokines, thus showing a great propensity to oxidative stress and inflammation [101]; therefore, excessive intra-abdominal fat accumulation may be the main determinant of MS. In a study consisting of 44 men and 61 women, correlation among visceral fat area, oxidative stress and MS has been proven, in which the visceral fat area was demonstrated to be the strongest and independent determinant of urinary excretion of a biomarker of systemic oxidative stress, associated with MS [102]. Furthermore, several studies utilizing cell culture and *ex vivo* tissue models strongly supported a causative role of oxidative stress in MS development [21,31]. In particular, Nox seems to play a central role in ethiology of obesity-related pathologies: studies carried out in obese mice demonstrated that inhibition of this enzyme reduces diabetes, adipokine deregulation, and hepatic steatosis [21].

ROS overproduction contributes to the onset of altered insulin sensitivity by several mechanisms. Oxidative stress activates stress signaling kinases, among which c-jun N terminal kinase 1 (JNK1) plays a key role in the aetiology of insulin resistance: by phosphorylating IRS-1 at inhibitory sites, JNK1 prevents recruitment of this protein to the activated insulin receptor and disrupts downstream events of the insulin signaling pathway. Alternatively, insulin resistance may derive from chronic inflammation promoted by oxidative stress. The pro-inflammatory cytokines TNFα and IL-6 have been shown to trigger phosphorylation of IRS-1, thus exerting an inhibitory action on insulin signaling; accordingly, genetic ablation either of TNFα or of its receptor can improve insulin resistance caused by obesity in rodent models [103]. Finally, oxidative damage to critical proteins in insulin-sensitive tissues could potentially affect their function and therefore the propagation of insulin-stimulated signals for instance, *in vitro* studies have shown that oxidative stress reduces the ability of insulin receptor to correctly bind insulin, and insulin resistance developed in older humans is often accompanied by reduced function of proteins involved in insulin signaling [31].

ROS accumulation also accounts for obesity-induced hypertension. Deregulation of insulin signaling leads to altered endothelial phosphatidylinositol 3-kinase (PI3K)/protein kinase B (Akt) pathway; therefore, endothelial cells lessen NO synthesis, so that decreased vasodilatation and increased blood pressure occur; moreover, activation of the renin-angiotensin system increases expression of mineral corticoid receptors in kidney, with subsequent sodium re-absorption and hypertension [96].

Redox-inflammatory processes, together with visceral adiposity, promote liver dysfunction, thus contributing to the onset of dyslipidemia, characterized by hypertriglyceridemia, high cholesterolemia (>240 mg/dL) and low concentration of HDL-cholesterol (<35 mg/dL) [31]; it is worth mentioning that drop in HDL levels worsens oxidative stress, as these lipoproteins also bind PON1 and transition metals [44].

### 3.3. Type 2 Diabetes

Type 2 diabetes (or non-insulin-dependent diabetes mellitus) develops in obese individuals as a result of hyperinsulinemia. Insulin-resistance stimulates pancreatic β-cells hyperplasia, in order to compensate for decreased hormone sensitivity; initial insulin overproduction is then followed by β-cell death and decreased insulin secretion. As β-cells possess a low scavenging capacity, they die because of chronic oxidative stress and adipokine secretion [32,104]; in the meanwhile, signaling coupling glucose metabolism with insulin secretion is impaired by oxidative unbalance. It has been recently shown that blood oxidative stress present in obese patients also damages insulin biological activity and contributes to the onset of immune responses to the hormone: indeed, human recombinant insulin incubated with whole blood derived from overweight or obese individuals shows increased polymerization paralleled by decreased hypoglycemic activity. In addition, insulin polymers generate new epitopes, thus triggering a new immunogenicity [66,71].

### 3.4. Cardiovascular Disease

Obesity (and especially abdominal fat) is often accompanied by increased risk of developing fatal and non-fatal CVD, including coronary artery disease, stroke, peripheral arterial disease, cardiomyopathy and congestive heart failure [105,106].

Circulating free fatty acids, together with insulin resistance, oxidative stress, mitochondrial dysfunction, endothelial dysfunction, and altered NO release, are key pathogenic factors [107]. Endothelial NO modulates vascular function; when its protective effects are impaired by oxidative stress, atherosclerosis and thrombosis will develop. Progression of vascular disease is further enhanced by obesity and hypertension that up-regulate Nox-derived ROS, renin-angiotensin system and cytokine release, thus generating a continuous vicious circle. ROS produced in the bloodstream stimulate growth of vascular smooth muscle cells, apoptosis of endothelial cells and migration of monocytes/macrophages, thus contributing to vascular inflammation and injury [108]. ROS (and decreased PON1 activity observed in obese subjects) also trigger LDL oxidation and macrophage activation, thus leading to foam cell and atherosclerotic lesion formation [54]. In addition, ROS and pro-inflammatory mediators, as well as low NO availability and high plasma levels of F2-isoprostanes, induce vasoconstriction and platelet hyperactivity [109].

Cardiovascular complications are also related to abdominal adiposity and high levels of several adipokines (leptin, resistin, chemerin, vaspin, visfatin, and omentin) [110]. Human aorta endothelial cells treated with chemerin show increased levels of mitochondrial ROS that mediate chemerin-induced autophagy [111,112]. Elevated leptin concentrations directly damage endothelial and vascular smooth muscle cells; moreover, they stimulate secretion by macrophages of the lipoprotein lipase, a pro-atherogenic enzyme enhancing monocyte adhesion, retention of lipoproteins in the sub-endothelial space and formation of foam cells [27].

Elderly individuals appear to be more vulnerable to obesity-associated vascular complications than younger individuals. Indeed, aging exacerbates obesity-induced inflammation in perivascular adipose tissue, which contributes to increased oxidative stress and inflammation in a paracrine manner [113].

### 3.5. Obstructive Sleep Apnea

Obstructive sleep apnea is a breathing disorder, characterized by repeated breathing arrests and intermittent hypoxia in sleep. This syndrome, which represents a risk factor for CVD, is often associated with central obesity, as 60%–90% of patients are obese. Visceral fat, oxidative stress, and inflammation are now recognized as pathogenetic mechanisms accounting for this respiration disorder [114]. High circulating levels of pro-inflammatory cytokines up-regulate NF-κB in neutrophils and monocytes of sleep apnea patients, thus enhancing ROS signaling and systemic inflammation in this pathology [115]. Finally, pauses in respiration change blood oxygen saturation, thus establishing an ischemia/reperfusion condition, which results in ROS overproduction [116].

### 3.6. Cancer

Oncogenesis is a multi-factorial phenomenon, in which redox unbalance plays a key role. Epidemiological studies have found a positive correlation between increased risk and worst cancer outcome and BMI and fat distribution [117,118]. Overweightness and obesity are responsible for 14% of cancer deaths in men and 20% of cancer deaths in women; obesity-related mortality is especially for prostate and stomach tumors in men, breast, endometrium, cervix, uterus, and ovaries in women and gastrointestinal tumors in both sexes [118]. The main determinants involved in carcinogenesis and cancer progression appear to be energy balance and calorie restriction, hyperinsulinemia, oxidative stress, and chronic inflammation, while relationship between MS and cancer is less well established.

Convincing evidence indicates that low or intermediate levels of oxidative stress cause DNA damage, which results in genomic instability associated with activation of oncogenes and/or inactivation of tumor suppressor genes. Altered gene expression may be achieved either by direct action of oxidative DNA modifications or may be mediated by epigenetic alterations; in particular, ROS have been shown to disrupt the epigenetic pattern, by producing carcinogens that induce hypermethylation and/or by regulating histone modifications and miRNA expression, therefore affecting tumorigenesis and cancer progression (extensively reviewed in Ref. [33]). The finding that insulin-resistant patients show an elevated risk for several types of cancer further support a link between oxidative stress/chronic inflammation and tumorigenesis; insulin signaling (together with insulin-like growth factor (IGF), leptin and inflammatory cytokines) has mitogenic properties through activation of the PI3K/Akt/mammalian target of rapamycin (mTOR) pathway [119]. Finally, accumulating evidence outlines the role of oxidative metabolites of estrogens in breast cancer, as their redox cycling is able to stimulate ROS generation causing DNA oxidative damage [33].

## 4. Potential Strategies to Reduce Oxidative Stress in Obesity

Strategies designed to increase antioxidant defenses in obese subjects could be useful to prevent and treat obesity co-morbidities. Herein, we will review recent experimental evidences on redox state modulation, in obesity and obesity-associated pathologies (Figure 1).

### 4.1. Weight Loss and Physical Activity

Weight loss associated with physical activity has been found to be the most efficacious approach to prevent dyslipidemia, hypertension, type 2 diabetes, CVD, NAFLD, and colorectal cancer [100,120,121], even though it is difficult to discriminate if observed effects are due to weight loss *per se* or specific diet administered to induce weight loss.

Among the positive effects of weight reduction in obese individuals, the best documented are decrease in oxidative damage and inflammation. Weight loss obtained via hypoenergetic diet influences ROS production, as indicated by specific markers of oxidative stress and proteins involved in mitochondrial-related oxidative processes. Different studies show that protein carbonylation, lipid peroxidation, oxLDL and 8-isoprostane, as well as inflammatory markers (such as C-reactive protein, IL-8 and genes involved in the TNF-α/NF-kB signal cascade), decline with weight reduction [122–126]. Metabolic functions ameliorate with reduction in oxidative stress, as evidenced by increased adiponectin levels and better liver function [127]. At a molecular level, lowering energy supply activates proteins of the sirtuin (SIRT) and Forkhead box, sub-group O (FoxO) families [128]: sirtuin are NAD^+^-dependent deacetylases that transcriptionally improve metabolic efficiency, strengthen antioxidant defenses and dampen inflammatory activities [129], while FoxO proteins modulate transcription of genes involved in energy homeostasis, cell survival and inflammatory responses, including NF-κB [130].

Diet-induced weight loss also reduces colorectal inflammation and greatly modulates inflammatory and cancer-related pathways; for instance, 10 obese premenopausal women showed TNF-α and IL-8 plasma levels significantly reduced after about 10% weight loss; in addition, rectosigmoid biopsies showed reduced inflammation markers and T cell and macrophage counts. Gene array experiments further confirmed the positive effects of weight loss on expression of inflammation- and cancer-related genes, including gene sets regulated by TNF-α, IL-6 and IL-17, prostaglandins, as well as redox-sensitive proteins, such as the signal transducer and activator of transcription 3 (STAT3), NF-κB, activating transcription factor (ATF) and cyclic AMP response element binding (CREB) [121].

Combination of hypoenergetic diet with regular exercise potentiates the beneficial effects on redox balance. Gutierrez-Lopez *et al.* [66] showed that hypocaloric diet plus regular moderate aerobic exercise was more effective than hypocaloric diet alone in decreasing oxidative stress markers and insulin polymerization, in 32 obese subjects. Rector *et al.* [131] reported that exercise-induced weight loss improved the whole body redox state, by modulating both oxidative (oxLDL, lipid hydroperoxides) and antioxidative (PON1) biomarkers. In obese children and adolescents, early lifestyle intervention (including both exercise and diet) appears to be the optimal approach to ameliorate endothelial dysfunction, inflammation, and oxidative stress [132]. Several studies proved evidence that health-beneficial effects of physical activity are independent from exercise-related changes in body weight [133]. In humans, exercise *per se* reduces Nox activity and ROS production, as well as blood pressure and inflammation [134]; it also exerts an anti-inflammatory action increasing anti-inflammatory cytokine (IL-1 and IL-10) levels and reducing generation of the pro-inflammatory cytokine TNF-α [135,136]. Therefore, regular physical activity appears to act as a natural antioxidant and anti-inflammatory strategy for preventing obesity-associated complications: it improves glucose-insulin homeostasis, endothelial function and antioxidant defenses, while lowering circulating triglycerides.

### 4.2. Dietary Pattern and Macronutrients

Some dietary components deeply contribute to oxidative stress; thereby reducing their content in food may be helpful to improve redox state, independently from weight reduction. Dietary factors, such as sugar-rich soft drinks and diet rich in SFA, have been shown to promote fat deposition and regain weight following weight loss [40,59]. High fructose consumption may lead to oxidative stress and metabolic alterations [137], whereas chronic consumption reduces insulin sensitivity and glucose tolerance, thus speeding up diabetes onset [138]. SFA have been shown to trigger lipotoxic mechanisms, both in experimental models and obese patients: indeed, SFA excess promotes hepatic steatosis and liver injury, by inducing ER stress, mitochondrial dysfunction, oxidative stress, and JNK signaling [139].

Mediterranean diet (rich in fruit, vegetables, whole-grains, legumes, nuts, fish, low-fat dairy products, moderate consumption of wine, and olive oil as principal source of fat) exerts preventive effects, being associated to significant reduction in overall mortality, incidence or mortality from CVD, from stroke and from cancer, Parkinson’s and Alzheimer’s diseases, and mild cognitive impairment [36,140–143]. By studying abdominally overweight men and women, van Dijk *et al.* [144] demonstrated that this kind of diet was able to reduce oxidative stress and inflammation. After eight weeks, they observed that participants who consumed Mediterranean diet had lower concentrations of circulating pro-inflammatory proteins and decreased gene expression of oxidative phosphorylation enzymes in peripheral blood mononuclear cells. A direct correlation between Mediterranean diet and protection against type 2 diabetes has recently been demonstrated in 7052 high cardiovascular risk subjects with genetic susceptibility to obesity and diabetes (genetic variants of *FTO* rs9939609 and *MC4R* rs17782313): when adherence to diet was low, carriers of the variant alleles had higher type 2 diabetes risk than wild-type subjects, but when adherence was high these phenomena disappeared. The study also reported that no interactions with macronutrients and food groups were found, suggesting that the overall dietary pattern was more important than specific foods or nutrients [145]. Conversely, other studies have found a positive effect of specific food consumption on redox balance and/or inflammatory biomarkers, endothelial function, insulin resistance, and occurrence of CVD or MS [146–151]. Evidence for a protective role of MUFA (present in significant amounts in olive oil) and ω-3 PUFA (present in significant amounts in fish) on CVD arises both from *in vitro* and *in vivo* studies. MUFA (high-oleic acid sunflower oil) replacement of SFA (palm oil) for eight weeks reduces metabolic stress, by decreasing oxidative phosphorylation in peripheral blood mononuclear cells, as well as plasma connective tissue growth factor and apoB concentrations in abdominally overweight men and women [144]. Eicosapentaenoic (EPA) and docosahexaenoic (DHA) acids can inhibit atherosclerosis by several mechanisms, including modulation of lipid metabolism and membrane fluidity, improvement of vascular endothelial function, inhibition of inflammatory processes, decrease of intracellular ROS and increase of antioxidant defenses [152]. An epidemiological survey performed on 65 rural counties in China showed a strong inverse correlation between erythrocyte DHA and CVD [153]. Low plasma EPA/arachidonic acid ratio was found significantly associated with a high prevalence of complex coronary lesions in patients with stable angina pectoris [154]. In fructose-fed rats, administration of dietary fish oil prevents endothelial dysfunction by increasing eNOS expression and decreasing oxidative stress [155]; accordingly, DHA has been proved to down-regulate TNF-α/NF-kB-mediated expression of adhesion molecules in endothelial cells [156]. A recent clinical trial reported a dose-dependent effects of fish oil (2, 4, and 6 g/day; 260 mg DHA and 60 mg EPA per gram of fish oil, for 12 weeks) on heart rate variability and arterial compliance in overweight and obese individuals [157]. In patients with type 2 diabetes mellitus, ω-3 PUFA (1.8 g EPA and 1.5 g DHA) supplementation for eight weeks positively affects platelet redox imbalance (reduced 8-isoprostane and superoxide levels) [158]. In obese glucose intolerant people, flaxseed supplementation (40 g/d for 12 weeks), supplying ω-3 PUFA (α-linolenic acid) and lignans (a polyphenol class), has been proved to decrease TBARs and insulin resistance [159]. An antiobesity effect of EPA has been reported recently in two distinct animal models of obesity; EPA treatment strongly suppressed body weight gain and obesity-related hyperglycaemia and hyperinsulinemia induced by HFHC diet, while reducing hepatic triglyceride secretion and changing very low density lipoproteins (VLDL) fatty acid composition. The role of ω-3 PUFA in preventing obesity and associated pathologies is further supported by Kusonoky *et al.* [160]: in 3T3-L1 adipocytes, EPA and DHA have an anti-oxidant effect via the Nrf-2/HO-1 pathway. Conjugated linoleic acids (CLA) are a family of isomers of linoleic acid (ω-6 PUFA), mostly present in meat and dairy products from grass-fed animals, which have received considerable attention because of their anti-obesity and anti-diabetic effects in certain animal models. These effects appear to be achieved through decrease in energy intake and lipogenesis, as well as increase in energy expenditure and lipid catabolism. Nonetheless, CLA effectiveness is quite controversial, since several studies failed to demonstrate significant effects on fat mass loss [161]. Furthermore, no beneficial effects on antioxidant defenses have been observed after eight weeks CLA supplementation in 29 healthy overweight/obese subjects; 2.4 g/day CLA mixture (36.9% of *cis*-9, *trans*-11 and 37.9% of *trans*-10, *cis*-12) did not change plasma TAC, lipid peroxidation, liposoluble vitamin concentrations, erythrocyte antioxidant enzyme activities, and leukocyte DNA damage [162].

### 4.3. Vitamins and Phytochemicals

Numerous studies have confirmed the strong association between diet rich in plant foods and health [41,151,163]; the positive effects of these foods may rely on their content on phytochemicals, antioxidant vitamins and fiber. Most of these dietary compounds contribute to a well redox balance by several mechanisms, such direct scavenging or neutralization of free radicals, modulation of enzyme activity and expression, and anti-inflammatory action. A diet with high total antioxidant capacity (an index of cumulative antioxidant capacity in foods) has been found inversely related to central adiposity, metabolic and oxidative stress markers, and risk for ischemic stroke [164,165]. In particular, a beneficial role of citrus fruits in obesity and obesity-associated diseases has been demonstrated by observational and clinical studies. Data from the National Health and Nutrition Examination Survey (*NHANES*) 2003–2006 show that squeezed orange juice consumers are 21% less likely to be obese and male consumers are 36% less likely to have MS, compared to non-consumers [150]. A positive effect of orange juice is further supported by the observation that this drink neutralizes post-prandial oxidative stress and inflammation induced by HFHC meal [166]. Mandarin juice daily consumption (500 mL of pure mandarin juice) positively affects antioxidant defenses and decreases oxidative biomarkers in obese children [167]. Other examples of useful plant foods are broccoli and carrots. Consumption of young broccoli sprouts improves oxidative stress in diabetic conditions: four weeks eating 10 g/day of broccoli sprouts powder significantly increases serum TAC and decreases oxidative stress index, MDA and oxLDL, in 21 patients with type-2 diabetes [168]. In 17 overweight subjects with elevated plasma cholesterol and triglyceride levels, drinking 470 mL of freshly squeezed carrot juice for three months (without changes in lifestyle) significantly increases TAC and decreases MDA in plasma; interestingly, this study reported that improvement on redox balance was not accompanied to decrease in cardiovascular risk markers, demonstrating that high consumption of a single plant food without lifestyle modifications is not sufficient to improve lipid profiles [148].

Antioxidant supplements (especially vitamin C, vitamin E, and carotenoids) may potentially benefit obese individuals. Observational studies showed that dietary antioxidant vitamins are inversely associated with CVD, cancer, and all-cause mortality; however, data from intervention trials are controversial [42–44,169]. Several short-term studies showed a positive role of vitamin E on oxidative stress and lipid metabolism: supplementation with natural vitamin E (800 IU daily for six months) decreased plasma 8-isoprostane levels in 80 overweight subjects [170]; α-tocopherol supplementation (300 IU daily for four months) provided clear benefits in reducing markers of oxidative stress and improving lipid state, in women with MS [171]. Vitamin E supplementation also seems to exert positive effects on breast cancer recurrence [172]. Conversely, other clinical trials failed to demonstrate positive effects of vitamin E supplementation on health, and adverse effects have also been reported. In women at high risk for cardiovascular events, vitamin E supplementation (600 IU daily, 9.4 years follow-up) had no effect on cancer incidence and death [43]; in addition, in healthy men vitamin E supplementation (400 IU of α-tocopherol daily; 7–12 years follow-up) increased prostate cancer incidence [173]. A recent systematic review of nine randomized controlled trials demonstrated no significant effect of vitamin E supplementation on glycemic control in type 2 diabetic patients and a modest improvement in glycosylated haemoglobin (HbA1c) was observed only in patients with high HbA1c or low serum vitamin E levels [174]. In patients with vascular disease or diabetes mellitus, long-term vitamin E supplementation (400 IU daily of natural vitamin E, seven years follow-up) did not prevent cancer or major cardiovascular events, and increased the risk for heart failure [175]. Furthermore, vitamin E supplementation (400 IU daily of synthetic α-tocopherol, eight years follow-up) was associated with increased risk of hemorrhagic stroke in a cohort of 14,641 US male physicians [176]. In light of the above evidences, routine vitamin E supplementation is not currently recommended in obese subjects.

High rate of vitamin C deficiency has been found in obese individuals and diabetic subjects [76,80]. In a cohort of middle-aged men and women enrolled in the EPIC-Norfolk study, higher plasma vitamin C levels were associated with a substantially decreased risk of diabetes [177]. Other prospective studies using the EPIC data reported an inverse association between plasma vitamin C levels and coronary artery disease [178], stroke [179], blood pressure [180], and heart failure [181]. It is important to mention that observational studies show some shortcomings and, therefore, they do not allow to univocally establish the role played by vitamin C on health outcomes; indeed, plasma vitamin C levels also reflect fruit and vegetable intake (so, the positive health effects may be not dependent on vitamin C content) and other lifestyle variables (such as socioeconomic position) positively related to health [182]. Data sustaining a beneficial role of vitamin C on primary and secondary prevention come from interventional studies. A positive role in diabetes prevention is supported by a large observational study reporting that vitamin C supplementation (500 mg daily) was associated with 9% reduction in prevalence of type 2 diabetes, in a population of older adults [183]. Vitamin C supplementation seems to be also useful on hypertension; indeed, a recent meta-analysis showed that a median intake of 500 mg per day reduced blood pressure in hypertensive and normal subjects [184]. On the other hand, other studies failed to ascertain effectiveness of vitamin C supplementation on reducing the risk of CVD [176] and cancer [43,185]. Although vitamin C is not toxic, available data do not sustain vitamin supplementation in obesity; however, it should be recalled that vitamin C deficiency is a common feature in obese individuals, therefore regular consumption of vitamin C-rich foods has to be recommended.

Carotenoids, widely distributed in plants, microorganisms and few animals, are powerful antioxidant compounds; some of them also show pro-vitamin A activity (especially β-carotene, α-carotene and β-cryptoxanthin). Obese people, and in particular those with high waist circumference, have low serum carotenoid levels [186]; consumption of carotene-containing vegetables and fruits (such as carrots, pumpkin, tomato, broccoli, spinach, pumpkins, apricots, and mandarins) has been found inversely associated with cardiovascular mortality and overall mortality in the elderly [187]. *In vitro* and animal studies show that carotenoids may exert a protective role in MS, CVD and cancer, acting as antioxidant and anti-inflammatory agents [188,189]. Data from the Coronary Artery Risk Development in Young Adults (CARDIA) study show that serum total and individual carotenoids (with the exception of lycopene) are inversely associated with markers of inflammation, oxidative stress, and endothelial dysfunction; furthermore, the sum of four carotenoids (α-carotene, β-carotene, lutein/zeaxanthin, cryptoxanthin) was inversely associated with hypertension incidence [190,191]. Despite these observations, the majority of supplementation studies failed to demonstrate a beneficial effect of β-carotene (the carotenoid most used in supplementation) on CVD, MS [45], and cancer risk [43]; enhanced risk for lung cancer and fatal coronary heart disease has also been reported [44,192]. Supplementation studies performed with other carotenoids show a positive action of these compounds on redox balance, inflammation and hypertension. Astaxanthin (a carotenoid produced by microscopic small algae and present in salmon, crabs, and lobster) supplementation (5 mg daily for three weeks) suppresses lipid peroxidation and stimulates activity of antioxidant defenses, in overweight and obese adults [193]. Supplementation of highly concentrated β-cryptoxanthin (approximately 4 mg daily for three weeks) improves serum adipokine profiles in moderately obese postmenopausal women [194]. Lycopene supplementation (15 mg for eight weeks) significantly decreases systolic blood pressure from the baseline value, in mildly hypertensive subjects [195]. Finally, a recent meta-analysis investigating the role of lycopene in type 2 diabetes mellitus showed that lycopene or tomato intake decreased oxidative stress in diabetic patients without decreasing the risk for having diabetes [196].

Polyphenols constitute the most abundant phytochemicals provided by food of plant origin, being widely distributed in fruits, vegetables, whole cereals, coffee, cacao, and tea. Estimated total polyphenol intakes are about 1000 mg/day in European populations [197]. In recent years numerous *in vitro* and animal studies have provided evidence that polyphenols may be protective against oxidative-triggered pathologies, including CVD, metabolic disorders, cancer, and obesity [41,198]; nonetheless, molecular targets related to pathogenic processes have not yet been fully characterized. These molecules show high antioxidant activity *in vitro*, but the biological relevance of this activity *in vivo* is questionable, because of low degree of absorption and rapid metabolism within the body. It is now well known that polyphenols exert their biological effects through other mechanisms, so that it should be better to consider them as “bioactive” compounds rather than “antioxidant” compounds [199]. The antioxidant activity of polyphenols relies on their ability to act as scavenger molecules, as well as to inhibit ROS-generating enzymes (including Nox and iNOS) and to enhance expression of antioxidant enzymes (including those responsible for glutathione synthesis and phase II drug metabolism, trough regulation of the Nrf2/Keap1 pathway). Polyphenols may have anti-obesity, anti-inflammatory, anti-diabetic, and anti-cancer properties through multiple mechanisms: they act by modulating inflammation and redox state, by regulating adipocyte differentiation and lipid metabolism, by inhibiting pancreatic lipase activity and intestinal permeability, and by interacting with gut microbiota [200–202]. Nonetheless, human prospective studies are lacking and a causal relationship between polyphenols, obesity and chronic diseases cannot be established [203]. Few short-term clinical trials investigated the role of polyphenol supplementation (single molecules or food extracts) in obesity and obesity-related diseases, by evaluating oxidative stress and inflammation markers, as well as glucose tolerance and CVD risk factors, and almost all found a positive role (see reference [204] for further details). Just an example, in healthy volunteers with high metabolic risk (first-degree relatives of type 2 diabetic patients), 2 g/day of grape polyphenols reduced high-fructose-induced oxidative stress and insulin resistance [205].

Although general mechanisms of action can be identified, nonetheless each polyphenol may exert distinct physiological effects, depending on its chemical structure, bioavailability and metabolism. According to their chemical structure, polyphenols are classified into different categories: phenolic acids, stilbenes, flavonoids (flavonols, flavanols, anthocyanins, flavanones, flavones, flavanonols, and isoflavones), chalcones, lignans and curcuminoids. Herein, we list some examples among the most studied and promising molecules.

Ferulic acid, a phenolic acid present in whole wheat, chocolate, apples, oranges, oregano, and sage, has been proved to be effective against high fat-induced hyperlipidemia and oxidative stress, via regulation of insulin secretion and regulation of antioxidant and lipogenic enzyme activities [206].

Resveratrol, a stilbene primarily found in red grapes, apples, and peanuts, can be useful to counteract obesity, metabolic disorders, CVD, and cancer, through multiple actions: it increases mitochondrial activity, counteracts lipid accumulation, decreases inflammation, improves insulin signaling and modulates redox balance. In obese subjects (BMI between 30 and 40), 28 days supplementation of resveratrol triphosphate (300 mg daily) decreases biochemical parameters of oxidative stress and modulates the expression of redox-sensitive genes in blood cells [207]. In mature human adipocytes, resveratrol reduces the expression of inflammatory mediators (TNF-α, IL-6, COX-2) and inhibits NF-κB signaling; it also induces lipolysis and apoptosis, while reducing lipogenesis and proliferation [41,208]. In pre-adipocytes, resveratrol down-regulates PPAR-γ expression (a nuclear hormone receptor, regulating adipocyte differentiation, insulin sensitivity and obesity susceptibility) and increases expression of genes modulating mitochondrial activity (SIRT3, uncoupling protein 1, mitofusin 2) [209]. Comparable metabolic effects have also seen in humans: a randomized double-blind crossover study in healthy obese men shows that resveratrol supplementation (150 mg daily) for 30 days lowers oxidative stress and mimics the effect of calorie restriction [210]. Several clinical studies showed that resveratrol supplementation improves insulin sensitivity in type 2 diabetes patients and decreases oxidative stress; it should be recalled that different amounts (ranging from 10 mg to 2 g) have been used, therefore it is not clear the absolute resveratrol concentration that could be effective in diabetes improvement [211,212]. In patients with stable coronary artery disease, daily consumption of a resveratrol-containing grape supplement (8 mg resveratrol) for one year provided a cardiovascular benefit, by increasing serum adiponectin, preventing plasminogen activator inhibitor-1 (PAI-1) increase and inhibiting atherothrombotic signals, in peripheral blood mononuclear cells [213]. In type 2 diabetes and hypertensive patients with coronary artery disease, one-year supplementation with a resveratrol-containing grape supplement (8 mg resveratrol) modulated inflammatory-related mRNAs and cytokine expression, in peripheral blood mononuclear cells [214]. Another positive action of resveratrol in obesity relies on its ability to reduce oxidative and inflammatory responses induced by HFHC meal: this effect is achieved by decreasing Nox activity and inducing NQO1 and glutathione *S*-transferase-1P expression in mononuclear cells [215], as well as by preventing oxidative stress-mediated intestinal barrier disruption [216]. Anti-cancer activity of resveratrol is supported by different pre-clinical and clinical studies; in healthy adults, repeated administration of high doses of resveratrol (2.5 g daily for 29 days) decreases circulating IGF-I and IGFBP-3 levels [217]. In adult women at increased breast cancer risk, resveratrol supplementation (5 or 50 mg trans-resveratrol, twice daily for 12 weeks) decreases methylation of the tumor suppressor gene RASSF-1α [218].

Quercetin, a flavonol present in apples, onions, scallions, broccoli, apples, and teas, is known to have multiple biological functions, including anti-inflammatory, anti-oxidative and anti-mutagenic activities. Quercetin attenuates adipogenesis in L6 myotubes, while in obese mice it decreases inflammation and improves insulin sensitivity, by increasing GLUT4 expression and decreasing JNK phosphorylation, as well as TNF-α and iNOS expression in skeletal muscle [219]. In primary human adipocytes, it prevents insulin resistance and down-regulates inflammation, by attenuating IL-6, IL-1β, IL-8 and MCP-1 expression [220]. Quercetin supplementation (10 mg/kg) lessens inflammatory state in the adipose tissue of obese Zucker rats and improves dyslipidemia, hypertension and hyperinsulinemia. Quercetin also lowers circulating glucose, insulin, triglycerides, and cholesterol levels in mice and rats fed a calorie-rich diet, and enhances adiponectin expression and secretion [204]. In overweight-obese subjects, 150 mg daily quercetin supplementation for six weeks significantly decreases plasma oxLDL and TNF-α [221].

Catechins are the most abundant flavonols contained in tea; they are also present in cocoa, grapes, and red wine [197]. In rats, catechins significantly lower oxidative stress and inflammation, by increasing expression of catalase and SOD and by decreasing Nox, iNOS, TNF-α and NF-κB activities [222]. Furthermore, 16 weeks epigallocatechin gallate (EGCG; 1 mg/kg) supplementation elevates thermogenesis, improves glucose tolerance and increases PPAR-γ expression, in rats feeding a high-fat diet [204]. In streptozotocin-diabetic rats, eight weeks EGCG (25 mg/kg) administration establishes a hypoglycemic condition paralleled by a better lipid profile [195]. Epicatechin-enriched diet reduces IGF-1 levels and prolongs lifespan in diabetic mice; similar results have also been found in *Drosophila melanogaster* [223]. In humans, catechins have been proven to ameliorate blood pressure, LDL-cholesterol, obesity, and CDVD risk factors. Catechin-rich beverages (green tea containing about 600 mg catechins) improve obesity and glycaemia in type 2 diabetes patients [224]. Daily supplementation of 379 mg green tea extracts reduces blood pressure, inflammatory biomarkers, and oxidative stress, and improves parameters associated with insulin resistance in obese, hypertensive patients [225]. Green tea beverage consumption (four cups daily) or extract supplementation (two capsules/day), both containing equal amounts of total catechins (900 mg), for eight weeks, significantly decrease body weight and lipid peroxidation, in obese subjects with MS [226]. Epidemiologic evidence suggests that green tea may also play a protective role on some type of cancers, as proposed by a recent *in vitro* study showing that EGCG inhibits proliferation of breast cancer cells, via down-regulation of the estrogen receptor-α gene [227].

Isoflavones (genistein, daidzein, and glycitein) are present in legumes, grains, and vegetables, but soybeans are the most important source of these polyphenols in human diet. Thanks to their chemical structure, they are able of exerting estrogen-like effects; for this reason, they are also classified as phytoestrogens and are considered useful towards hormone-dependent cancers (in particular, prostate and breast cancers) [228]. Long-term safety studies suggest that women consuming an isoflavone-rich diet may have a lower risk of endometrial and ovarian cancers [229]. Recent evidences suggest that these compounds also protect against obesity and co-morbities [230]; their anti-adipogenic and anti-lipogenic effects may be due to the ability of estrogen receptors to interact with PPAR, thus modulating adipose development, insulin sensitivity and fatty acid metabolism. Other protective mechanisms are reduction of oxidative stress and inflammation, genistein seems a promising candidate to counteract detrimental effects of ROS, as it acts as an antioxidant by chelating metals and by increasing both reduced/oxidized glutathione ratio (GSH/GSSG) and mitochondrial membrane potential. Moreover, genistein restores antioxidant enzyme activities and decreases production of ROS and pro-inflammatory cytokines, as well as iNOS and endothelial NOS (eNOS) contents. In rats feeding a high fat diet, it inhibits inflammation and progression of NAFLD, by activating JNK and inhibiting NF-κB, as well as TNF-α and IL-6 secretion [231]. In postmenopausal women, genistein supplementation (>1 mg/day) decreases BMI, body fat mass, waist size, and increases blood HDL [232,233]; accordingly, six months isoflavone supplementation (80 mg daily) in healthy women decreases DNA oxidative damage and increases plasma TAC [234]. One serving/day of isoflavone-enriched pasta (33 mg total isoflavones), for eight weeks, increases plasma TAC and GSH levels, while decreasing oxLDL and isoprostane 8-*iso*-PGF_2α_, in overweight diabetic subjects [235].

Curcumin, a principal curcuminoid extracted from turmeric (a spice derived from the rhizomes of Curcuma longa), has anti-cancer, anti-inflammatory, anti-obesity, and anti-diabetic properties [236]. The underlying mechanisms of action seem to involve regulation of redox-sensitive transcription factors, inflammatory cytokines and growth factors. At cellular level, curcumin may induce a mild oxidative and metabolic stress, leading to adaptive responses characterized by increased antioxidant (catalase, MnSOD, HO-1) and lipid metabolism (aP2/FABP4, CD36, HMG-CoA reductase, CPT-I) enzymes [237]. In liver, curcumin prevents high fat diet-induced insulin resistance and obesity, attenuating lipogenesis [238]. In adipose tissue, it inhibits macrophage infiltration and NF-κB activation [239]. In muscle, by activating Nrf2, it ameliorates oxidative stress and glucose tolerance [240]. Finally, curcumin has a well-recognized anti-cancer activity that, at least in some cases, may be due to its estrogen-like effects [241].

Capsaicinoids and capsinoids, alkaloids primarily found in red hot peppers and sweet peppers, exert pharmacological and physiological actions, including anti-cancer, anti-inflammatory, antioxidant, and anti-obesity effects [242]. It has been reported that capsaicinoid consumption increases energy expenditure and lipid oxidation, reduces appetite and energy intake, thus promoting weight loss [243]. The molecular mechanisms of action remain not fully understood, although it is now established that stimulation of the transient receptor potential vanilloid type-1(TRPV1) is responsible for much of the observed effects [243]. Capsaicin also attenuates obesity-induced inflammatory responses by reducing TNF-α, IL-6, IL-8, and MCP-1 levels [244], while enhancing adiponectin levels, important for insulin response [245].

### 4.4. Aminoacids

Among new promising strategies to prevent and treat adiposity and the associated metabolic syndrome, supplementation of specific aminoacids deserves particular mention. Arginine supplementation reduces white adipose tissue in Zucker obese rats and obese patients with type II diabetes; the mechanism of action relies on stimulation of gene expression, especially of PPAR-γ coactivator 1 (the main regulator of mitochondrial biogenesis) [246], eNOS, HO-1 and AMP-activated protein kinase (AMPK). l-arginine also increases blood flow, lipolysis, and glucose and fatty acid catabolism; in the meanwhile, it inhibits fatty acid synthesis, thus improving metabolic profile [247]. Another positive aminoacid is leucine that stimulates protein synthesis and attenuates adiposity (by increasing fatty acid oxidation and mitochondrial biogenesis in muscle and adipocytes). Nutraceutical supplementation (2.25 g leucine and 30 mg pyridoxine) three times/day, for four weeks without energy restriction, increases fat oxidation and reduces insulin resistance, oxidative stress and inflammation biomarkers, in overweight or obese subjects [248].

### 4.5. Gut Microbiota

Recent evidence shows quantitative and qualitative differences in gut-microbiota among subjects with high and low risk developing obesity and obesity-related complications (such as insulin resistance, type-2 diabetes, MS and NAFLD). Microbiota transplantation studies performed both in animals and humans suggest that gut “dysbiosis” by itself can cause weight gain and insulin-resistance [38,249]. The hypothesized mechanisms include: increased efficiency of energy uptake from food, modification of gut permeability, release of gut hormones, induction of oxidative stress and inflammation [250]. Microbial composition of gut microbiota is influenced by body weight and diet components (such as fiber, polyphenols and lipids). In obese individuals, weight loss decreases *Firmicutes to Bacteroidetes* ratio [251], whereas a high fat diet and caloric load increases it [252]. High relative abundance of *Firmicutes* is associated with metabolic endotoxemia, due to increased absorption of lipopolysaccharide (LPS), which reaches circulation and induces oxidative stress, inflammation and impairment of insulin signaling [38,250]. Actually, several studies report that probiotics (live micro-organisms, especially *Bifidobacterium* and *Lactobacillus*, conferring a health benefit for the host) and prebiotics (non-viable food components, especially inulin-type fructans, conferring a health benefit for the host associated to modulation of microbiota) can regulate obesity-host metabolism, in obesity and obesity-related disorders [37,38,40,250]. A study performed on rodents, to investigate the effect of novel probiotics on high-fructose-induced MS, underlines the ability of gut microbiota to ameliorate oxidative stress. Animals feeding a high-fructose diet develops clinical features of MS, including high plasma glucose, insulin, triglycerides, total cholesterol and oxidative stress levels, as well as increased liver mass and lipids compared to chow fed controls; probiotic treatment (*L. curvatus* HY7601 and *L. plantarum* KY1032) lowers almost all these parameters and reduces lipogenesis via down-regulation of SREBP1, FAS and stearoyl-CoA desaturase-1 mRNAs, and increases β-oxidation via up-regulation of PPAR-α and CPT-2 mRNAs [253].

Several studies underline the positive role of probiotics also in modulation of redox state. In type 2 diabetic patients, 300 g/day of probiotic yogurt (containing *Lactobacillus acidophilus La5* and *Bifidobacterium lactis Bb12*) consumption ameliorates blood glucose and antioxidant parameters (including TAC, as well as SOD and GPx activities) and decreased oxidative markers (MDA) [254]. Furthermore, probiotics counteract exercise-induced oxidative stress: four weeks, daily consumption of a mixture of two probiotic strains (*L. rhamnosus IMC 501* and *L. paracasei IMC 502*) increases plasma TAC levels and decreases plasma ROS metabolites [255]. In healthy subjects, daily consumption of 150 g of goat milk fermented with *Lactobacillus fermentum ME-3* for three weeks increases TAC and decreases oxidative markers (oxLDL, 8-isoprostanes and GSSG/GSH ratio), in human blood and urine [256].

Ultimately, it is now evident that gut microbiota can play a key role on bioactivity of polyphenols and the presence of different polyphenol-metabolizing microbes can, at least in part, explain the inter-individual variability on health effects observed upon polyphenol intake; at the same time, polyphenols, by changing intestinal redox state, may control microbiota sub-populations, therefore, microbiota-poliphenols interlink represents an additional check-point of oxidative-stress-mediated pathologies [202].

## 5. Conclusions

Since 1980, overweightness and obesity has doubled worldwide and are now increasing in low- and middle-income countries, principally in urban settings. The National Center for Chronic Disease Prevention and Health Promotion (CDC) reported that more than one-third of U.S. adults (35.7%) were obese, in 2009–2010, while in European Union countries, obesity affects 23% of women and 20% of men [257]. In addition, inadequate dietary patterns and low levels of physical activity have sharply increased worldwide childhood obesity: in 2010, more than 40 million children under five were overweight.

Obesity represents a major risk factor for severe pathologies (including NAFLD, diabetes, coronary heart disease, hypertension, stroke, and cancer); this implies increased morbidity and mortality rates [1,257–259], as well as high health care costs. Several strategies have been developed by WHO, International Obesity Task force (IOTF), and CDC, for the prevention and control of obesity, especially concerning lifestyle intervention (physical activity and diet). A lot of research is currently aimed at characterizing obesity-induced molecular changes and genetic susceptibility to obesity-associated diseases, in order to identify potential molecular targets for preventive strategies. What is emerging from the available data is that obesity is strictly linked to changes in redox state: oxidative stress mainly results from positive energy balance that leads to excessive fat accumulation in non-adipose tissues, with subsequent development of obesity co-morbidities. Moreover, among obese people, some individuals are particularly vulnerable, as demonstrated by recent nutrigenetic studies [145,260].

Cross-sectional, prospective and intervention studies have investigated the effects of weight loss, physical activity, dietary pattern and specific food components on redox balance. Weight loss associated with physical activity seems to be most effective approach to reduce oxidative stress and risk for complications in obese individuals [100,120,121]. High energy, SFA/trans-fatty acid- and fructose-rich diets appear to be related to oxidative stress and specific pathologies, including NAFLD and CVD [261]; therefore consumption of such kind of food should be restricted. Relevance of Mediterranean diet on health has recently been demonstrated in genetically predisposed individuals: Ortega-Azorín *et al.* show that, when adherence to the Mediterranean diet pattern was low, the obesity risk alleles were often associated to type-2 diabetes, regardless of BMI [145].

As obesity is linked to oxidative stress, antioxidant supplementation should be useful to prevent or slow down progression of associated pathologies. Although several investigations have underlined the ability of vitamin C, vitamin E, and β-carotene supplementation to improve oxidative stress, inflammation and metabolic risk factors [42,170–172], nonetheless these are short-term studies carried out on a small sample and evaluating few biomarkers; conversely, long-term prospective studies showed no effects or negative outcomes on prevention of type 2 diabetes, CVD, cancer, and overall mortality [43,44,176]. However, it should be recalled that lack of effects may depend on experimental limitations, including: lack of a suitable control group (control subjects likely continued to eat vitamin enough to meet requirements), dose and type of supplement used (for example, synthetic α-tocopherol instead of a mixture of α-, β-, γ-, δ-tocopherols), duration of trials (the follow-up period should not be sufficient to cover the critical etiologic window), criteria for selection of patients (participant ages, co-morbidities, antioxidant state at baseline), and other confounding factors acting throughout the lifespan [182]. Based on this background, supplementation with antioxidant compounds (alone or in combination) is actually not recommended for MS, CVD, and cancer prevention.

Polyphenols are thought to exert a positive action on obesity and prevention of chronic diseases, not only by modulating redox state, but also thanks to their anti-estrogenic or estrogenic effects, anti-inflammatory activity and ability to regulate gut permeability and microbiota composition [41,199,204]. In the last years, scientific research has been focused on specific molecules (such as lycopene, resveratrol and EGCG), for which many human intervention studies are now available [196,211,218,225,226]. Data from these studies are promising, even if they should be interpreted carefully, especially considering the short follow-up period and the great heterogeneity of doses and type of supplements used (food extract or chemically isolated molecules).

Probiotic supplementation is emerging as a novel strategy to improve redox state, via reduction of LPS-induced endotoxemia associated to microbiota dysbiosis and modulation of polyphenols bioavailabiliy and bioactivity [37,202,249]. Nonetheless, the gut microbiota profile is profoundly influenced by body weight and food components (including fiber and saturated fatty acids), thus making probiotic supplementation ineffective if it is not accompanied by changes in dietary habits. Finally, it must be regarded that different bacterial strains exert different effects on health promotion, most of which are not fully elucidated; therefore, recommending probiotic supplementation to prevent obesity or related-pathologies seems still premature.

In conclusion, obesity-associated oxidative stress and diseases can be reduced mainly by weight loss together with physical activity; therefore, this should be the first and more important goal to be achieved. Secondly, obese subjects should obtain a great benefit from a regular consumption of foods exerting positive effects on health (including modulation of redox state), such as fruits and vegetables (rich in antioxidant vitamins, phytochemicals, and fiber), tea (rich in cathechins), spices (such as curcumin and red hot pepper), fish (rich in ω-3 PUFA), and low-fat dairy products (especially those rich in probiotics).
